# Ultrasonic Lens Based on a Subwavelength Slit Surrounded by Grooves

**DOI:** 10.3390/s140508821

**Published:** 2014-05-19

**Authors:** Vicente Gomez-Lozano, Pilar Candelas, Francisco Belmar, Constanza Rubio, Antonio Uris

**Affiliations:** Centro de Tecnologías Físicas, Universitat Politécnica de Valencia, Camino de Vera s/n. 46022 Valencia, Spain; E-Mails: vgomez@fis.upv.es (V.G.-L.); pcandelas@fis.upv.es (P.C.); fbelmar@fis.upv.es (F.B.); crubiom@fis.upv.es (C.R.)

**Keywords:** subwavelength slit, ultrasonic lens, grooves array

## Abstract

The lensing capabilities of a single subwavelength slit surrounded by a finite array of grooves milled into a brass plate is presented. The modulation of the beam intensity of this ultrasonic lens can be adjusted by varying the groove depth. Numerical simulations as well as experimental validations at 290 kHz are shown. The experimental results are in good agreement with the numerical simulations. This system is believed to have potential applications for medical ultrasound fields such as tomography and therapy.

## 1. Introduction

Since Ebbesen *et al.* [1] discovered the phenomenon of extraordinary optical transmission in metal films perforated with two-dimensional subwavelength hole arrays, linked with the excitation of surface plasmons, there has been a growing interest in exploring the optical properties of subwavelength holes and slits [2–7]. The ability of a single subwavelength slit perforated on metallic film surrounded by grooves to enhance light transmission as well as its beaming effect has attracted great attention [8–11].

Motivated by the wave nature of light and sound, the focus of research on electromagnetic waves has been transferred to acoustic waves, taking into account that the electromagnetic wave is a transverse vector while the acoustic wave is a scalar longitudinal wave. In recent years, the study of acoustic transmission through subwavelength apertures has received a lot of attention. The sound transmission through one and two dimensional aperture arrays has been studied theoretically [12,13] and confirmed experimentally for slit [14] and holes arrays [15–18]. On the other hand, the feasibility of sound collimation through a plate with a subwavelength slit surrounded by a finite array of grooves was demonstrated theoretically [19] and confirmed experimentally using a bull's eye structure [20]. In this sense, several theoretical studies have demonstrated the beaming capability of a subwavelength slit surrounded by a finite array of grooves [21,22]. Moreover, the fact that the evanescent waves of subwavelength apertures coupled with Fabry-Perot resonances inside an aperture causes an efficient transmission of the evanescent waves has openned a new avenue in acoustic metamaterials. The capability of acoustic imaging of these metamaterials based on subwavelength apertures is the reason for this amount of attention during these last years [23–26].

The purpose of this work is to show, through both simulations and experiments, that a brass plate with a single subwavelength slit surrounded by a finite array of grooves acts as an ultrasonic lens and the beam intensity modulation can be shifted by varying only the groove depth, maintaining the other parameters such as groove width and groove period fixed.

## 2. Experimental Section

**Figure 1.**
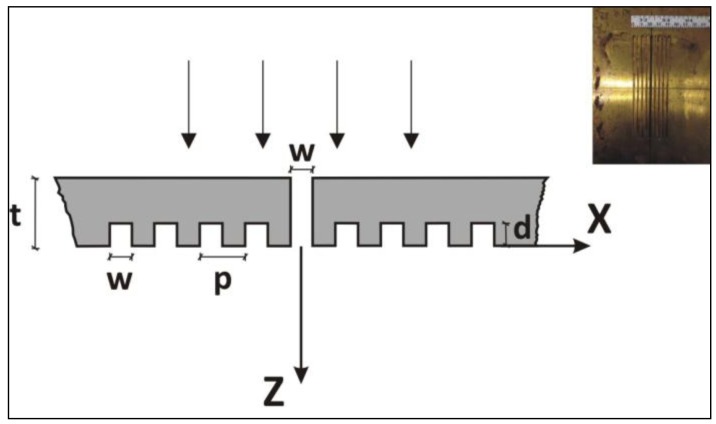
Schematic diagram of the corrugated plate. The inset shows a picture of the plate.

The basic structure considered is a brass plate with a subwavelength center-slit surrounded by a symmetric array of 4 grooves on each side of the slit at the output surface, as depicted in Figure 1. Preliminary numerical calculations showed that more than four grooves did not enhance the results. The thickness of the plate is *t* = 5 mm and the period of the grooves is *p* = 5 mm. The width of the central slit and the grooves is *w* = 2.5 mm. Three different depths of the grooves are considered, *d* = 1, 2 and 3 mm.

**Figure 2.**
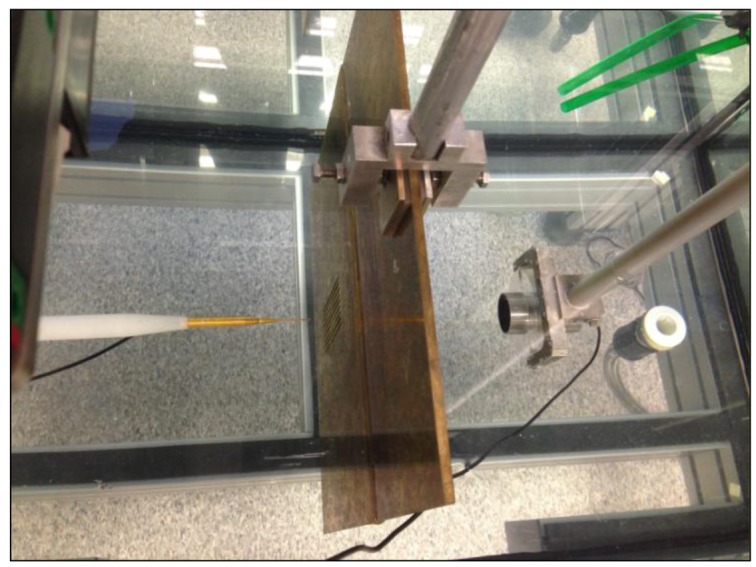
Picture of the experimental set-up.

The experimental set-up is based on the ultrasonic immersion transmission technique. The sample was placed in a water tank and the alignment and positioning were provided by an automated positioning system built around a water tank, which is capable of sweeping the hydrophone through a 3D grid of measurement points located at any trajectory inside the tank. Two different kinds of transducers were used: a piston transducer (Imasonic, Les Savourots, France) centered at a frequency of 250 kHz with an active diameter of 32 mm was employed as an emitter and a Polyvinylidene fluoride (PVDF) needle hydrophone (model HPM1/1, Precision Acoustics Ltd., Dorchester, UK) with a diameter of 1.5 mm and with ±4 dB bandwidth spans from 200 kHz to 15 MHz as a receiver. The pulse launched by the emitter piston transducer through the inspected plate was detected by the hydrophone and acquired by the pulser/receiver, post-amplified and digitalized by a digital PC oscilloscope (Picoscope model 3224, Pico Technology, St Neots, UK). Time domain data were finally analyzed after averaging 100 different measurements. The time window was set to be wide enough to collect only direct transmission through the plate and to avoid indirect transmission due to lateral reflections. Scanning was done using the automated positioning system along a plane normal to the plate, with a spatial resolution of 0.5 × 0.5 mm^2^. Figure 2 shows an image of the experimental set-up.

## 3. Results and Discussion

**Figure 3.**
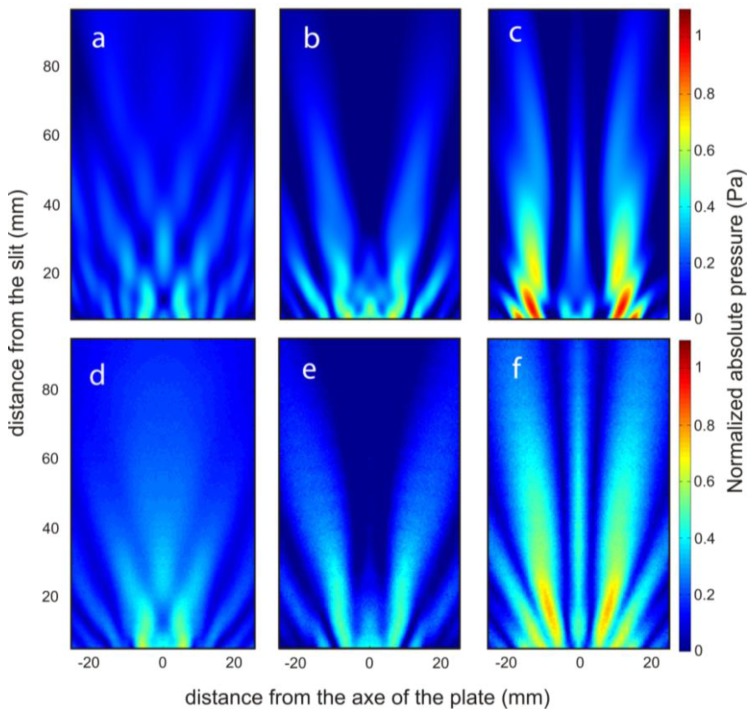
The normalized absolute pressure field maps in the *XZ* plane of the corrugated plates considered are shown. Simulated results for a groove depth of (**a**) 1 mm, (**b**) 2 mm and (**c**) 3 mm. Experimental results for a groove depth of (**d**) 1 mm, (**e**) 2 mm and (**f**) 3 mm.

**Figure 4.**
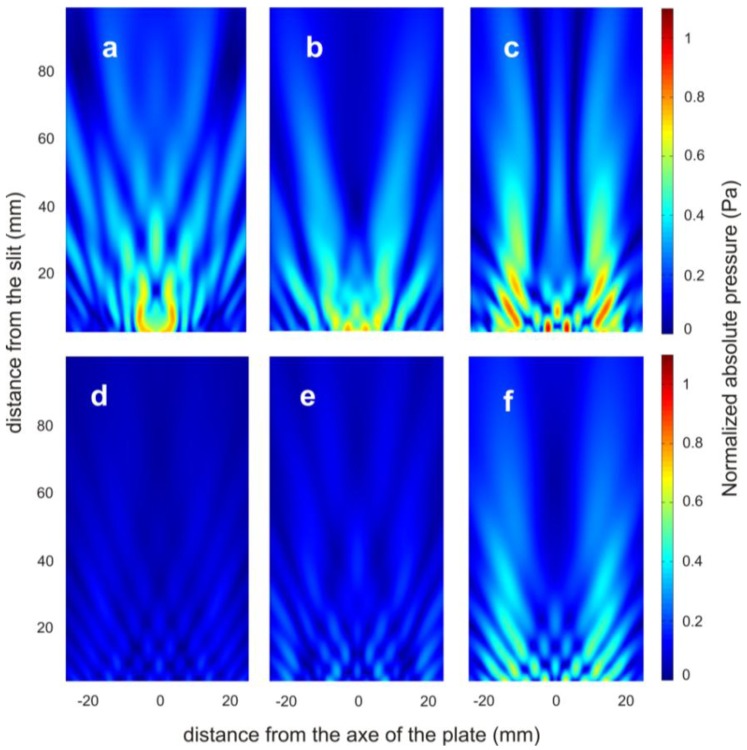
The normalized absolute pressure simulated field maps in the *XZ* plane of groove-grating with central slit plate for a groove depth of (**a**) 1 mm, (**b**) 2 mm and (**c**) 3 mm and groove-grating without central slit plate for a groove depth of (**d**) 1 mm, (**e**) 2 mm and (**f**) 3 mm.

The sound pressure maps of the transmission field were simulated by using the commercial software COMSOL Multiphysics 3.5.a (Comsol AB., Stockholm, Sweden). In the simulations, the brass plate was modelled as an elastic solid and the Helmholtz equation for the fluid medium was used together with the usual boundary conditions in the solid/fluid interface. More details about the model equations and boundary conditions can be found in reference [27]. The free field conditions for the outgoing waves were modelled using perfect matched layers [28]. In the simulations, the density, the Young's modulus and the Poisson ratio of the brass plate were set at 8490 kg/m^3^, 120 × 10^9^ N/m^2^ and 0.31, respectively. The water density, the bulk and dynamic viscosities values were set at 1000 kg/m^3^, 2.47 × 10^−3^ Pa·s and 0.89 × 10^−3^ Pa·s, respectively. By way of example, the numerical simulation and experimental pressure field maps at 290 kHz are presented in Figure 3a–f. These pressure maps involve dynamic scattering processes of two types of waves [21]: the acoustic surface evanescent waves due to the interface of a periodically grooved subwavelength structure and the cylindrical waves which are due to the imaginary point source at the end of the slit related to the well-known Huygens' Principle. The in-plane propagation of the acoustic surface evanescent waves depends on the groove width (*w*), groove depth (*d*) and the period of the grooves (*p*) by [29]:

(1) $$k_{x}=k{\left(1+\frac{w^{2}}{p^{2}}\tan^{2}kd\right)}^{1/2}$$

(2) $$k_{z}=jk\frac{w}{p}\tan\left(kd\right)$$

where *j* = (−1)^1/2^, *k* is the wavenumber and *k_x_* and *k_z_* are the respective *X* and *Z* wavenumber components. The cylindrical wave is governed by the thickness of the structure (*t*) and is related to the Fabry-Perot resonances. The overlapping of both the cylindrical waves and the acoustic surface evanescent waves produces the modulation intensity of the beam. In such a way, the mere presence of groove-grating does not allow the modulation of the beam because the structure only radiates evanescent waves, so the pressure amplitude decays quickly away from the structure surface [30]. To verify this, absolute pressure simulated field maps of groove-grating with central slit (Figure 4a–c) and groove-grating without central slit (Figure 4d–f) plates are plotted. Three different groove depths are considered: 1 mm (Figure 4a,d), 2 mm (Figure 4b,e) and 3 mm (Figure 4c,f).

**Figure 5.**
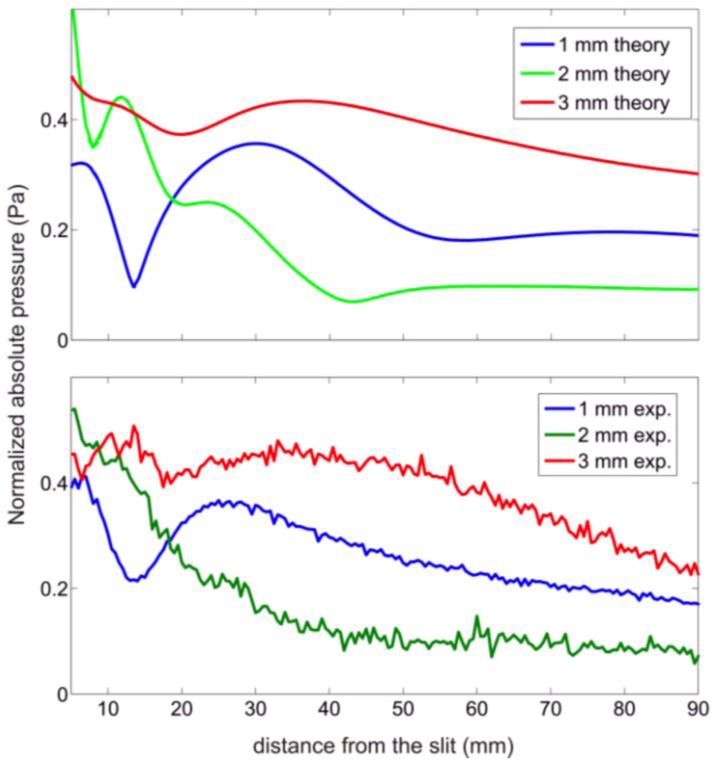
Pressure field at the line *x* = 0 of the corrugated plates considered. (**a**) Simulated results and (**b**) Experimental results.

To quantify the pressure at a certain distance for different groove depths, it is necessary to cut the pressure field maps at *x* = 0. Figure 5 shows these results both numerically and experimentally. As the interest is in the far-field region, where the edge effects can be neglected, only the results from 20 mm to the slit are taken into account. In this region, maintaining the transducer power constant, and depending on the grooves̶ depth at a certain distance from the slit, different pressure values are obtained as observed in Figure 5. The experimental results are in close agreement with the simulated ones.

## 4. Conclusions

In conclusion, both the simulated and experimental ultrasonic beaming effects of a single subwavelength slit surrounded by a finite array of grooves have been shown. The experimental results are in close agreement with the simulated ones. This modulation phenomenon can be explained as an interference phenomenon. At the output side, the slit diffracts a beam into free space and through acoustic surface evanescent waves to grooves. A single groove has cavity modes that depend on the groove depth and width.

Each groove couples acoustic surface evanescent waves into other grooves and radiative waves into free space when the phases are matched. The constructive interference of the acoustic waves emerging from the slit and grooves will increase the pressure at a point where the phase difference between the waves is a multiple of 2π. The phase difference depends on the depth of the grooves, and thus the far-field pressure distribution can be modified. This device is believed to have potential applications for medical ultrasound fields such as tomography and therapy.
